# Reply to Mirabelli et al. Is Mesothelioma Unrelated to the Lung Asbestos Burden? Comment on “Visonà et al. Inorganic Fiber Lung Burden in Subjects with Occupational and/or Anthropogenic Environmental Asbestos Exposure in Broni (Pavia, Northern Italy): An SEM-EDS Study on Autoptic Samples. *Int. J. Environ. Res. Public Health* 2021, *18*, 2053”

**DOI:** 10.3390/ijerph18137181

**Published:** 2021-07-05

**Authors:** Silvia Damiana Visonà, Silvana Capella, Sofia Bodini, Paola Borrelli, Simona Villani, Eleonora Crespi, Andrea Frontini, Claudio Colosio, Ruggero Vigliaturo, Elena Belluso

**Affiliations:** 1 Unit of Legal Medicine and Forensic Sciences, Department of Public Health, Experimental and Forensic Medicine, University of Pavia, 27100 Pavia, Italy; sofia.bodini01@universitadipavia.it; 2 Department of Earth Sciences, University of Torino, 10125 Torino, Italy; silvana.capella@unito.it (S.C.); ruggero.vigliaturo@gmail.com (R.V.); elena.belluso@unito.it (E.B.); 3 Interdepartmental Center for Studies on Asbestos and Other Toxic Particulates “G. Scansetti”, University of Torino, 10121 Torino, Italy; 4 Unit of Biostatistics and Clinical Epidemiology, Department of Public Health, Experimental and Forensic Medicine, University of Pavia, 27100 Pavia, Italy; paola.borrelli@unich.it (P.B.); simona.villani@unipv.it (S.V.); 5 Laboratory of Biostatistics, Department of Medical, Oral and Biotechnological Sciences, University “G. d’Annunzio” Chieti-Pescara, 66100 Chieti, Italy; 6 Occupational Health Unit, Santi Paolo e Carlo Hospital, 20142 Milano, Italy; eleonora.crespi@asst-santipaolocarlo.it (E.C.); claudio.colosio@unimi.it (C.C.); 7 Department of Life and Environmental Science, Polytechnic University of Marche, 60131 Ancona, Italy; a.frontini@univpm.it; 8 Department of Health Sciences, University of Milan, 20122 Milano, Italy

We appreciate very much the interest of Mirabelli et al. in our work [1] and for giving us the opportunity to clarify some points and, especially, the background and context of our conclusions.

First, we would like to alleviate the doubts raised by the authors about the correct interpretation of the papers cited in the manuscript.

Wagner et al. [2], in the results section, stated that “the patients with mesotheliomas and controls had similar absolute amounts of crocidolite in their lungs, and although in percentage terms the lung of the patients with mesotheliomas contained more crocidolite than those of the controls, this difference could easily been due to chance (*p* > 0.2)”. This sentence means, undeniably, that the authors compared, among a group of asbestos-exposed textile workers, malignant mesothelioma (MM) cases with people who died of other causes (including lung cancer and other types of cancer). They did not find a statistically significant difference in lung crocidolite concentration among these two groups. What Mirabelli et al. [3] report in their comment refers to other findings, which are certainly true. Wagner et al. found much higher concentrations in the lungs of asbestos textile workers (considering the whole group, including those with MM and other causes of death) compared to the general population (“historical controls”) not exposed to asbestos. In our manuscript, we do not deny that lung samples taken from a population of individuals occupationally exposed to asbestos contained more asbestos compared to a random series of lung samples (without any known exposure).

In Rogers’ study [4], the authors compared the lung content in patients with MM with people without any asbestos-related disease. They found, indeed, a dose–response relationship. Similar results were also pointed out by Sakai et al. [5], who analyzed 16 MM and 16 controls (known to be exposed to asbestos and not, respectively) using analytical transmission microscopy. In our introduction we clearly stated that these two studies found a higher concentration of asbestos in subjects with MM compared to controls. Therefore, we disagree with the claimed “misquoting” of such articles.

Concerning the efficacy of environmental exposure, citing Barbieri et al. [6] and Magnani et al. [7], we merely meant to underline the importance of this kind of exposure. These works, indeed, pointed out high concentrations of asbestos in individuals who were not exposed occupationally (not unlike what we detected in occupationally exposed subjects in our study, using the same technique as Barbieri et al. [6]). This point is important because environmental exposure is still present worldwide, and it is probably more frequent than occupational exposure, due to the large diffusion of asbestos artifacts, and it is important to be aware of the effects of this kind of asbestos exposure in terms of lung fiber burden. We do agree with Mirabelli et al. when they highlight that environmental exposure in Broni and Casale Monferrato in the 1950s and 1980s was particularly high.

We only stated that conclusions about the link between asbestos concentrations in lungs were inconsistent for two reasons. On the one hand, the different techniques used make it difficult to compare the results and, on the other hand, even though the study cited above [4,5,8] showed a dose–response relationship, in other studies this relation was not observed [9,10,11,12]. In 1984, Churg et al. analyzed the lung content of six long-term chrysotile miners and millers with pleural MM and controls (miners and millers without asbestos-related diseases), finding similar lung burdens and similar dimensional characteristics of fibers in cases and controls, but MM patients presented more components of chrysotile ore (chrysotile and tremolite asbestos) [9]. McDonald et al. conducted electron microscopy observations on lung samples of Quebec miners, revealing a similar amount of chrysotile in MM and controls and attributed most MM cases to amphiboles [13]. At the same time, Morinaga et al. found asbestos in 19 of 23 examined MM cases [10]. Amphiboles were detected in 13 cases, while in five cases only chrysotile was found. Five out of the 17 controls’ lungs contained asbestos fibers. Another electron microscopic study on lung content conducted on 126 autoptic samples (divided into MM, lung cancers, asbestosis and normal lungs) concluded that the concentration of chrysotile was similar among the groups, whereas the amphibole concentration shows higher levels in MM and asbestosis compared to normal lungs and lung cancer patients [11]. Likewise, a 1994 study on autoptic lung samples of shipyard and insulation workers (exposed to chrysotile and amosite) evidenced significantly higher levels of amosite in asbestosis patients compared to subjects without asbestos-related diseases, but failed to identify a correlation between asbestos concentration in lungs (and concentration of each kind of asbestos) and MM [12].

Regarding fibers with a length <5 µm (that is erroneously reported by Mirabelli et al. as “length >5 mm” and “diameter <0.5 mm”), we decided not to consider them in this study according to the widely accepted definition of fiber [14]. Yet, we also detected and analyzed fibers shorter than 5 µm, as with scanning electron microscopy (SEM) this is definitely achievable. None of the short fibers identified as asbestos (that were present in about 40% of cases) were classified as chrysotile/asbestiform antigorite.

Concerning the identification of fibers with diameter <0.5 µm, we underline that the technique used in our paper is the same that was used in two previous papers (in which Mirabelli, together with Belluso and Capella, was the co-author) about asbestos lung content in rats [15,16]. In both of them, some fibers identified as chrysotile/asbestiform antigorite were detected: therefore, it is very unlikely that chrysotile has gone undetected due to technical issues.

In addition, during observation of the samples, every time a fiber was not well observable at 2000×, we increased magnification (in order to obtain better images and more reliable measures), as the SEM has a resolution of 0.2 µm. This means, again, that chrysotile was, indeed, absent and not just undetected. This statement is in good agreement with the time passed between the last exposure and death of our 72 subjects (8–44 years), long enough to explain a complete chrysotile clearance.

The previous statement is further corroborated by preliminary investigations on some of the samples used in our study, which we have been carrying out recently by TEM energy dispersive spectroscopy (EDS) and selected area electron diffraction (SAED) (unpublished data). In the samples so far analyzed with this technique, the absence of chrysotile has been confirmed, whereas amphibolic fibers were found.

The analysis of the concentration of asbestos bodies (ABs) in organic samples and, in particular, in human lung tissue is essential for the study of asbestos-related diseases and for the evaluation of past exposure. In the literature, there are several different methods, and this makes the data hardly comparable with each other.

The Biofibre Group has prepared and described in detail a shared method of preparation and analysis of human lung tissue for the determination of the concentration of ABs in optical microscopy. The method is also applicable to the analysis of biological fluids (bronchioloalveolar lavage, sputum).

This validated method is convenient from a time and cost point of view, and is certainly reliable, but we chose to use SEM also for ABs quantification because the main goal of the study (as clearly specified in the manuscript) was to quantify, measure, and classify asbestos fibers (not detectable at optical microscopy). Therefore, it was much more expensive and time-consuming for us to prepare two samples for each subject and conduct two different and separate analyses, one using optical microscopy and one with SEM. Besides, as SEM allows the counting and visualization of asbestos bodies, there was no reason to perform an additional analysis using an optical microscope.

Regarding the last point raised by Mirabelli et al., we are well aware that the samples taken from asbestosis patients cannot be regarded as “controls”, because they died from an asbestos related disease. Regardless, we believe that comparing individuals who died from MM to others who died from asbestosis, who were both exposed to asbestos in similar settings, can provide useful information. In fact, much exposure to asbestos is required to develop asbestosis. We are interested in understanding why some individuals, so heavily exposed that they had asbestosis, did not develop MM and if there is any difference between these two groups in terms of concentration and type of asbestos in their lungs. We could easily have used controls (individuals from the general population without any asbestos-related disease) but we believe that the results, in that case, would not have been very informative, because it is obvious that MM patients (whose exposure to asbestos is very well documented) are likely to have higher levels of asbestos in their lungs compared to the general population. Moreover, an unrelated series of samples from the general population had already been analyzed by Capella et al. [17] and showed asbestos in low concentrations.

We are not discussing whether asbestos causes MM, as this has already been incontrovertibly demonstrated. We are trying to understand more about the biological events that take place in the lungs of individuals who, after asbestos exposure, develop MM and in those who do not, and if there is any difference in the lung response against asbestos in MM patients compared to heavily exposed individuals who inhaled so much asbestos that they develop asbestosis. We found that, in the analyzed series of 72 individuals, in MM cases there were fewer fibers and fewer ABs compared to asbestosis patients. This does not question the role of asbestos in causing MM, but allows new considerations beside what is well known. Moreover, a non-negligible proportion of MM patients show no asbestos in their lungs.

It is also important to remember that the detected fiber burden in lungs is not the exact expression of the fibers that were inhaled by the subjects. As already specified above, 8–44 years passed between the end of exposure and death of individuals analyzed in our paper. It is interesting to notice any difference between asbestosis and MM because it might reflect different responses of the lung microenvironment to asbestos. In particular, as we found no asbestos in some MM patients and no chrysotile at all, we have to focus on the role of chrysotile that has been degraded in the lung. In fact, if they have had amphiboles, we would have observed them under SEM-EDS. Perhaps the process of fragmentation or engulfment of fiber fragments by macrophages has a detrimental role that might contribute to triggering carcinogenesis. Asbestosis patients, compared to MM, had more ABs, which may contain chrysotile that has been covered instead of removed. We cannot be sure about the mineralogic nature of fibers inside ABs, as it is not possible to analyze the inner part of them using SEM-EDS, and therefore we cannot distinguish between chrysotile and other kinds of asbestos. Maybe, in patients with asbestosis, the capability to cover fibers (especially chrysotile) might be important as a protective mechanism against the generation of free radicals and oxidative stress, which could contribute to causing cancer. The role of the covering process in preventing the formation of free radicals of oxygen has already been suggested by previous experimental studies [18,19].

The results pointed out by our study call for more research in this field, and namely not only observational studies on human samples, but also experimental studies in cultured lung and mesothelial cells. Regarding lung samples, we are currently working on another series of deceased subjects exposed only to chrysotile in order to understand more about the clearance and the effects of this kind of asbestos on the lung and pleural microenvironment.
